# Maximizing cost-effectiveness by adjusting treatment strategy according to glaucoma severity

**DOI:** 10.1097/MD.0000000000005745

**Published:** 2016-12-30

**Authors:** Ricardo Augusto Paletta Guedes, Vanessa Maria Paletta Guedes, Carlos Eduardo de Mello Gomes, Alfredo Chaoubah

**Affiliations:** a Statistics Department and Public Health Department, Federal University of Juiz de Fora; b Ophthalmology Department, Santa Casa de Misericórdia Hospital; c Glaucoma Department, Paletta Guedes Ophthalmological Center, Juiz de Fora, MG, Brazil.

**Keywords:** cost-effectiveness analysis, laser treatment, medical treatment, primary open-angle glaucoma, quality of life, trabeculectomy

## Abstract

**Background:**

The aim of this study is to determine the most cost-effective strategy for the treatment of primary open-angle glaucoma (POAG) in Brazil, from the payer's perspective (Brazilian Public Health System) in the setting of the Glaucoma Referral Centers.

**Methods:**

Study design was a cost-effectiveness analysis of different treatment strategies for POAG. We developed 3 Markov models (one for each glaucoma stage: early, moderate and advanced), using a hypothetical cohort of POAG patients, from the perspective of the Brazilian Public Health System (SUS) and a horizon of the average life expectancy of the Brazilian population. Different strategies were tested according to disease severity. For early glaucoma, we compared observation, laser and medications. For moderate glaucoma, medications, laser and surgery. For advanced glaucoma, medications and surgery. Main outcome measures were ICER (incremental cost-effectiveness ratio), medical direct costs and QALY (quality-adjusted life year).

**Results:**

In early glaucoma, both laser and medical treatment were cost-effective (ICERs of initial laser and initial medical treatment over observation only, were R$ 2,811.39/QALY and R$ 3,450.47/QALY). Compared to observation strategy, the two alternatives have provided significant gains in quality of life. In moderate glaucoma population, medical treatment presented the highest costs among treatment strategies. Both laser and surgery were highly cost-effective in this group. For advanced glaucoma, both tested strategies were cost-effective. Starting age had a great impact on results in all studied groups. Initiating glaucoma therapy using laser or surgery were more cost-effective, the younger the patient.

**Conclusion:**

All tested treatment strategies for glaucoma provided real gains in quality of life and were cost-effective. However, according to the disease severity, not all strategies provided the same cost-effectiveness profile. Based on our findings, there should be a preferred strategy for each glaucoma stage, according to a cost-effectiveness ratio ranking.

## 1 Introduction

Glaucoma is the leading cause of irreversible blindness in the world. Knowing its associated costs is very important for planning actions to decrease the economic and social impact of blindness.^[1,2]^ Glaucoma leads to elevated direct (recurrent consultations, frequent ancillary tests, chronic use of medications, surgery, etc.) and indirect costs (temporary or permanent absence from work).^[3–5]^

Disease severity is a major driver for glaucoma-related costs. Literature shows that healthcare costs associated with glaucoma tend to increase when the diagnosis is made in late stages of the disease.^[3–5]^

Health technology assessment is an important and useful tool due to variability of clinical practice, uncertainty about the actual impact of health interventions, rapid development, and diffusion of new technologies and incompatibility between new and established technologies. The availability of new health technologies raises questions about how to best allocate limited resources.^[6,7]^

There are different types of health economic evaluation studies, such as cost-minimization, cost-effectiveness, and cost-benefit studies. Cost-effectiveness studies assess both the costs and the effectiveness of a health intervention. When effectiveness is measured in quality-adjusted life-years (QALYs), a metric that incorporates both quantity and quality of life, some authors refer to it as a cost-utility analysis.^[6,7]^ The Brazilian Ministry of Health has encouraged more cost-effectiveness studies to improve the efficiency of the Brazilian Public Health System (SUS).^[7]^

There are several different strategies for treating glaucoma. In 2002, Realini and Fechtner^[8]^ described 56,000 different ways to treat glaucoma. In routine practice, first-line glaucoma therapy is usually medications.^[9]^ However, and recently, some authors have advocated the use of laser trabeculoplasty or filtering surgery as possible primary treatment strategies.^[9–15]^ Some studies have even suggested that initial laser therapy could save costs by postponing the use of eye drops.^[11–13]^

The aim of this study was to determine the cost-effectiveness of observation, medications, laser trabeculoplasty, and filtering surgery as primary treatment strategies in primary open-angle glaucoma (POAG) patients, according to disease severity, within the SUS.

## 2 Methods

A hypothetical cohort of POAG patients in treatment within the SUS comprised the study population. We divided the patients into 3 groups according to disease severity in early, moderate, and advanced glaucoma. Based on the Hodapp, Parrish, and Anderson criteria, we defined early glaucoma as a visual field mean deviation (MD) index >−6.00 dB; moderate glaucoma as an MD between −6.00 and −12.00 dB; and advanced glaucoma as an MD <−12.00 dB.^[16]^ The study setting was the SUS Glaucoma Referral Centers.

Costs perspective was that of the payer (SUS as payer for the medical services), according to the guidelines of the Brazilian Ministry of Health.^[7]^ We included in this analysis only the medical direct costs. We did not consider nonmedical direct and indirect costs.

The different treatment strategies analyzed were the following: observation, primary medical treatment, primary laser trabeculoplasty, and primary filtering surgery. We did not consider all strategies in all glaucoma stages. We decided to adapt the treatment strategy to the disease severity. For the early glaucoma group, we tested observation, medications, and laser. We did not include surgery here, because filtering surgery is not a common approach in early cases and there is some evidence in the literature that surgery at this stage can lead to a loss in patients’ reported quality of life.^[17]^ In moderate glaucoma, we assessed the following treatment strategies: medications, laser, and surgery. For the advanced glaucoma group, the treatment strategies were medications and surgery. We excluded at this stage the laser trabeculoplasty, because this kind of treatment is not advisable in late glaucoma stages, due to low efficacy in this group of patients (they need a very low target intraocular pressure [IOP]) and the risk of postlaser IOP spikes.

Study horizon was the mean life expectancy for the Brazilian population.^[18]^ Starting age of each patient cohort varied according to the disease stage. Early glaucoma patients’ starting age was 40 years. For the moderate and advanced glaucoma cohorts, starting age was 60 years. In all groups, we tested different starting ages as part of our sensitivity analysis. We applied a discount of 5% in both costs and effectiveness, following the guidelines from the Brazilian Ministry of Health.^[7]^

We used the QALY as our effectiveness measure for the interventions. QALY is a preference-based metric, which incorporates the concepts of quality and quantity of life, and derives from the utility values. Utility values for our analysis were those suggested by Brown et al^[19]^ and confirmed by Lee et al.^[20]^ These values were obtained using the time trade off method, through direct interviews with glaucoma patients in different disease stages.^[19]^

For this study, we obtained the costs for each intervention from the SUS procedures list (SIGTAP table).^[21]^ We followed the guidelines of the Ministry of Health for obtaining the usual frequency of medical visits and ancillary examinations for glaucoma patients.^[22]^ For the medication costs, we considered the prices the SUS pays the Glaucoma Referral Centers for medication reimbursements.^[22]^ Monetary values are in Reais (R$), the Brazilian national currency, and refer to the year of 2014. We also present the values in US dollars (US$), to allow international comparisons. Conversion exchange values refer to December 31, 2014 (1 US$ = 2.66 R$).

In the medical therapy strategy, we assessed the mean number of medications per patient and the proportion of the different classes of medications in each glaucoma stage through a cross-sectional evaluation of 225 consecutive glaucoma patients, seen by the authors. The only adverse event considered in this treatment strategy was the inappropriate use of beta-blocker in patients with asthma. Following the suggestion made by previous authors, we added 23.8% in the final mean cost per patient using this medication.^[11]^

Primary treatment with laser considered performing laser trabeculoplasty 360° in both eyes right after the diagnosis. We made no distinction between argon laser (ALT) and selective laser (SLT), and we allowed for 1 repeated treatment for each eye, if necessary. According to Cantor et al,^[23]^ we added 21.0% onto the cost of initial laser trabeculoplasty, to account for the possible repetition of the laser therapy. In subsequent years, we considered the costs for introducing glaucoma medications according to the literature (50% efficacy, patients without the need for medications, at the end of each successive year).^[11]^ We did not consider the costs of adverse laser events, such as transient uveitis or retinal detachment, due to their low incidence. Costs of inappropriate use of beta-blocker were also included in this treatment strategy, using the same approach described previously.

For the surgical treatment alternative, we considered newly diagnosed POAG patients submitted to a filtering procedure (trabeculectomy) in both eyes. We considered the possibility of another surgery, when necessary, by adding 20.0% of the surgical procedure cost to the initial surgery cost.^[11]^ We also took into consideration the higher rate of cataract surgery in operated glaucoma patients, by adding 20.0% of the phacoemulsification and intraocular lens implantation costs.^[11]^ Transient postoperative complications (shallow anterior chamber, bleb leaks, choroidals, etc) were not considered, and we did not account for endophthalmitis costs, due to its very low incidence. In this treatment strategy, we also allowed the introduction of glaucoma medications, when necessary. The proportions and frequency of glaucoma medications in operated POAG patients were derived and adapted from the literature.^[24]^ We also included the costs for the inappropriate use of a beta-blocker.

For the cost-utility analysis, we constructed three Markov models, 1 for each glaucoma stage. In model 1, patients with early-stage disease entered the model at 40 years of age. This model had 5 states: early glaucoma (entering state); moderate glaucoma; advanced glaucoma, bilateral blindness, and death (terminal state). Duration of the cycle was 1 year and participants had to enter this model in the early glaucoma state. After each year, they could remain in the same state or pass to the next state, in that previously described order, according to the transition probabilities. Participants could not go back to a previous state, nor skip states. However, they could achieve the death state from any of the other states, according to the Brazilian Life Tables.^[18]^ We tested the following interventions: observation, medications, or laser in this model. For models 2 and 3, we used the same rationale, except that participants entered the model at a different age (60 years in both models) and at a different state: moderate glaucoma and advanced glaucoma, respectively. Interventions assessed were medications, laser, and surgery in model 2, and medications and surgery in model 3. We obtained the transition probabilities for each of the study treatment strategies from the literature.^[11,25,26]^ The choice for Markov modeling was based on the characteristics of the disease: glaucoma is a chronic illness with recurrent costs, which affects patient quality of life.

As in any modeling study, we made several assumptions. Cycle duration was predetermined at 1 year. In model 1, starting age was 40 years, because it is after this age that both POAG incidence and prevalence increase.^[27]^ In the other 2 models, starting age was 60 years. We chose this age because this is the most common mean age in glaucoma studies and we believe this a good approximation to real life.^[28]^ Primary medical treatment strategy considered the same sequence of medications for all patients. Treatment started with prostaglandins, and, in case of failure of achieving target IOP, 0.5% timolol maleate and 2% dorzolamide were added in this order. We made this choice based on our clinical practice and according to the Brazilian Glaucoma Society guidelines.^[29]^ We decided to exclude the option of using 0.2% brimonidine, due to its approximately 20% incidence of allergy and its possible neuroprotection action that could interfere and change the transition probabilities.^[28,30]^ Apart from this, it has the same indications and the same efficacy as 2% dorzolamide.^[28]^ Laser strategy as primary therapy considered 360° trabeculoplasty in both eyes and a possible retreatment if necessary.^[11]^ In case of failure in controlling IOP after a repeated laser treatment, patients were allowed to use glaucoma medications, in the following order: prostaglandins, 0.5% timolol maleate, and 2% dorzolamide.^[11,29]^ For the surgical treatment strategy, primary filtering surgery was the first glaucoma therapy. We also accounted for the possibility of 20% of reoperations and a 20% increase in cataract surgery.^[11]^ In cases of surgical failure, we allowed the patients to use the following sequence of glaucoma medications: prostaglandins, 0.5% timolol maleate, and 2% dorzolamide.^[29]^

Another important assumption is that we used fixed transition probabilities in all models. We did not allow adjusting of the probabilities according to the evolution of the model. We also assumed utility values did not vary according to the different glaucoma therapies and therefore we applied the same utility values for all treatment strategies, following some evidence in the literature.^[31]^

The main outcome measure for this study was the incremental cost-effectiveness ratio (ICER), measured in R$/QALY and US$/QALY.

We tested the robustness of our models by performing univariate sensitivity analysis for all parameters, through a tornado analysis, and individually for those variables that had the most impact on model results.

We collected our data in Microsoft Excel 2013 (Microsoft Corp., Redmond, WA) and we performed the cost-effectiveness analysis using TreeAge Pro 2011 Health Care software (Tree Age Software, Williamstown, MA).

This study was approved by the Ethics Committee of the Federal University of Juiz de Fora and adhered to the tenets of the Declaration of Helsinki. Informed consent was not necessary because participants were hypothetical (Markov modeling).

## 3 Results

For all models, we considered 3 parameters: medical direct costs and utility values for each model state, and the transition probabilities between states. Tables 1 and 2 show the resources used and their associated costs, and also the costs for each model state for the different treatment strategies, respectively.

**Table 1:**
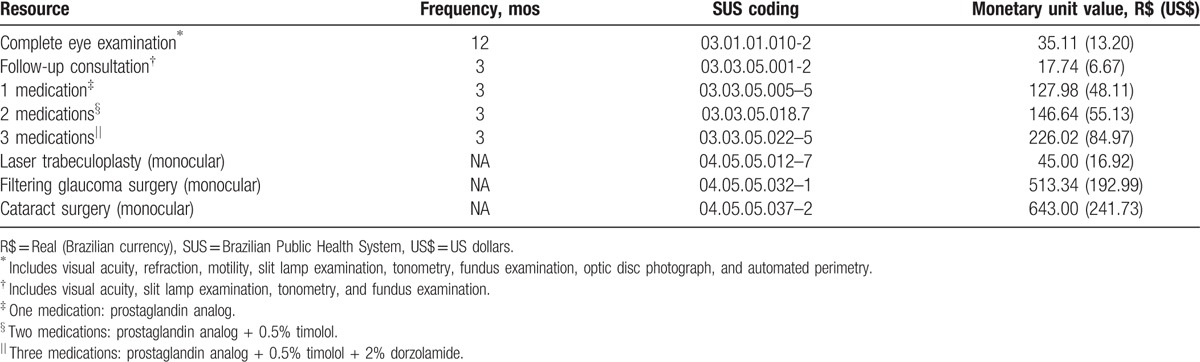
Resources use and their associated costs.

**Table 2:**
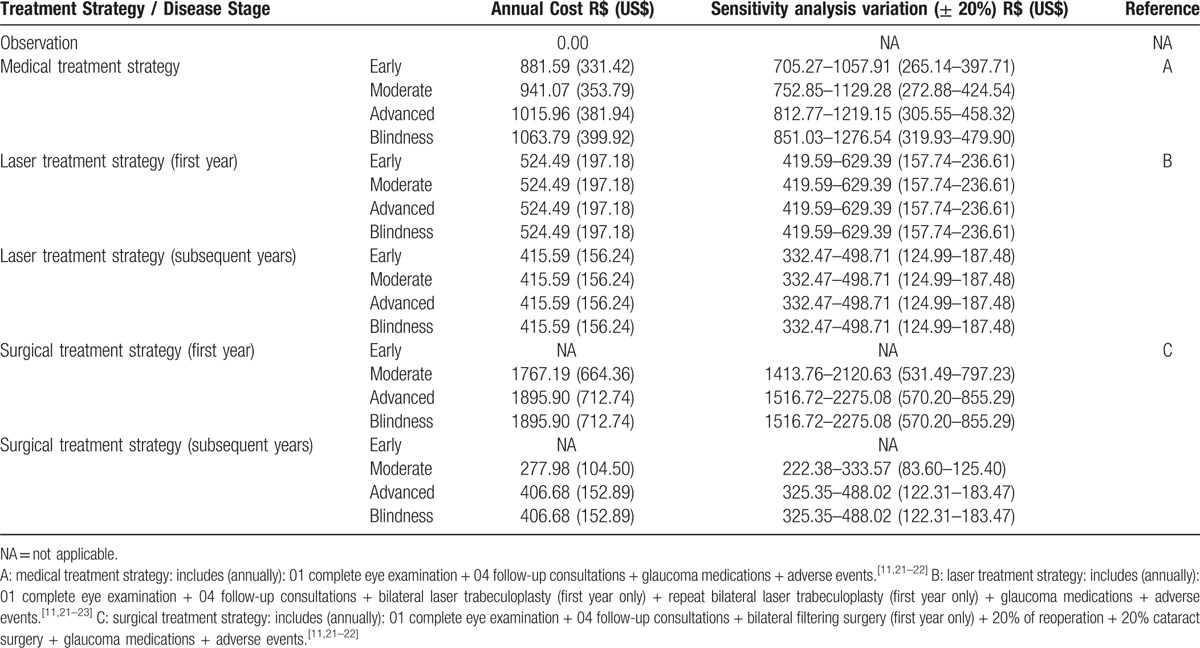
Direct medical costs for each model state according to treatment strategy.

In the medical treatment strategy, we obtained the proportion of each type of glaucoma medications for the different disease stages from a cross-sectional study of 225 consecutive POAG patients in treatment in our clinic. We found the following medication proportions for the early glaucoma stage: 53% of patients on 1 medication, 29% on 2 medications, and 19% on 3 medications. For moderate glaucoma, the proportions were 28% on 1 medication, 44% on 2 medications, and 28% on 3 medications. In the advanced glaucoma stage, 23% of patients were on 1 medication, 31% on 2 medications, and 46% on 3 medications. For bilateral blindness patients, 9% were on 1 medication, 36% on 2 medications, and 55% on 3 medications.

In the laser strategy, the proportion of patients on medications was 50% each year. Out of those 50%, half of them (25%) were considered to be on 1 medication and the other half on 2 medications. We did not consider differences in proportions according to glaucoma stage.

In the surgery strategy, we obtained the proportions of medication use from the literature. For moderate glaucoma, 75% of patients were off medications. Ten percent, 12%, and 3% of the patients were on 1, 2, and 3 medications, respectively. For the advanced glaucoma stage, 59% of patients were off drugs, and 17%, 14%, and 10% were on 1, 2, and 3 medications, respectively. We used these same proportions for bilateral blindness patients.

The utility values (and the deterministic variation for the sensitivity analysis) for each glaucoma stage (model state) were as follows: early glaucoma: 0.92 (0.80–0.99), moderate glaucoma: 0.89 (0.70–0.95), advanced glaucoma: 0.86 (0.60–0.90), bilateral blindness: 0.26 (0.20–0.60), and death: 0.00. Table 3 presents the transition probabilities between model states.

**Table 3:**
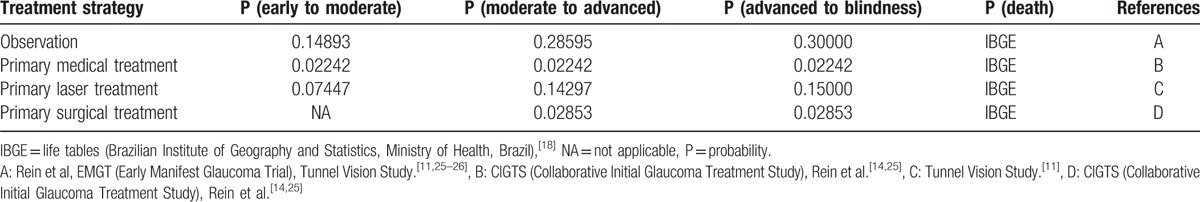
Transition probabilities between health states.

We present the results concerning the total medical direct costs, effectiveness, and cost-effectiveness analysis for each model in Table 4. In model 1, both primary laser treatment and primary medical therapy are cost-effective strategies, with a better ICER favoring primary laser. In model 2, both primary laser and surgery were cost-effective; however, surgery led to similar costs with a better effectiveness profile. Medical therapy strategy for moderate glaucoma had the highest cost-effectiveness ratio. In the advanced glaucoma model, the least expensive alternative was surgery, but medical treatment was also a cost-effective option.

**Table 4:**
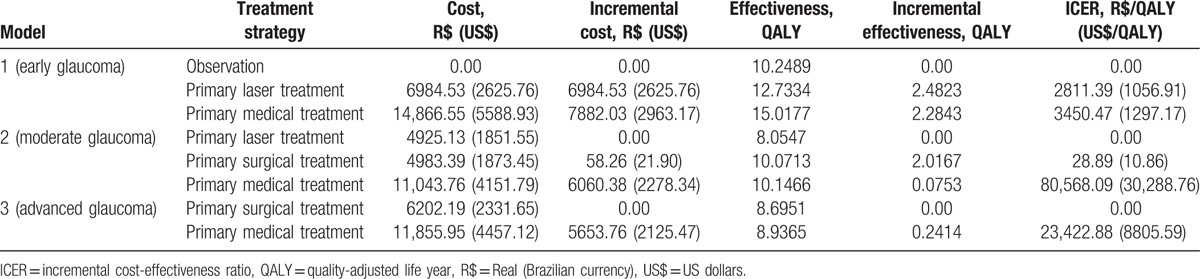
Medical direct costs, effectiveness, and cost-effectiveness analysis for the different glaucoma treatment strategies for each glaucoma model.

**Figure 1:**
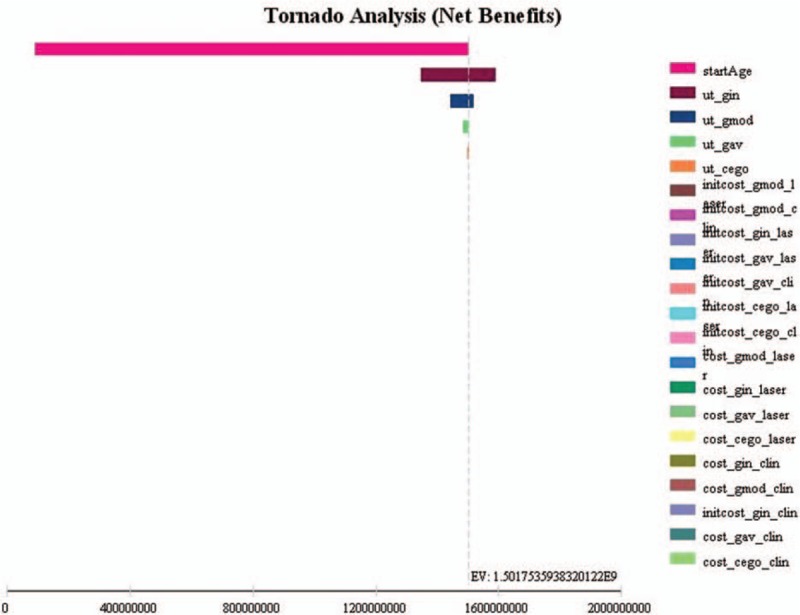
Model 1 (early glaucoma) sensitivity analysis (Tornado analysis) of all parameters.

**Figure 2:**
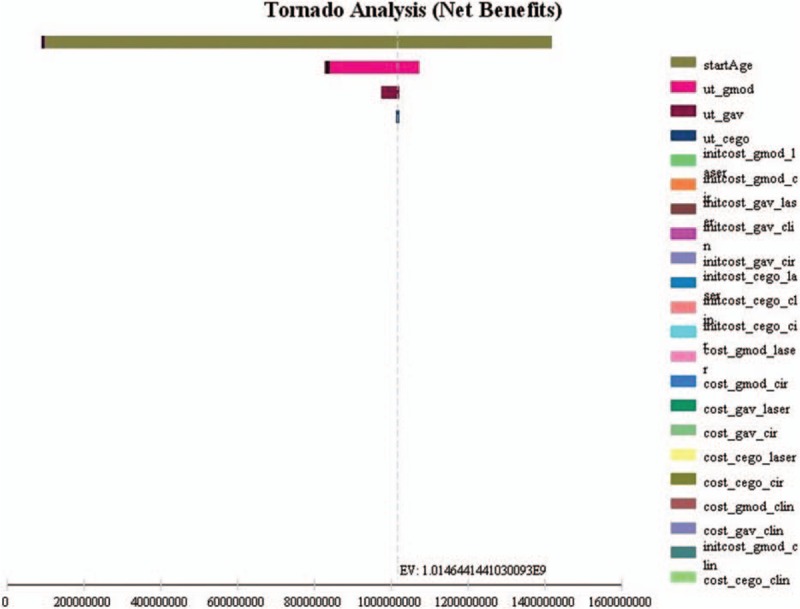
Model 2 (moderate glaucoma) sensitivity analysis (Tornado analysis) of all parameters.

**Figure 3:**
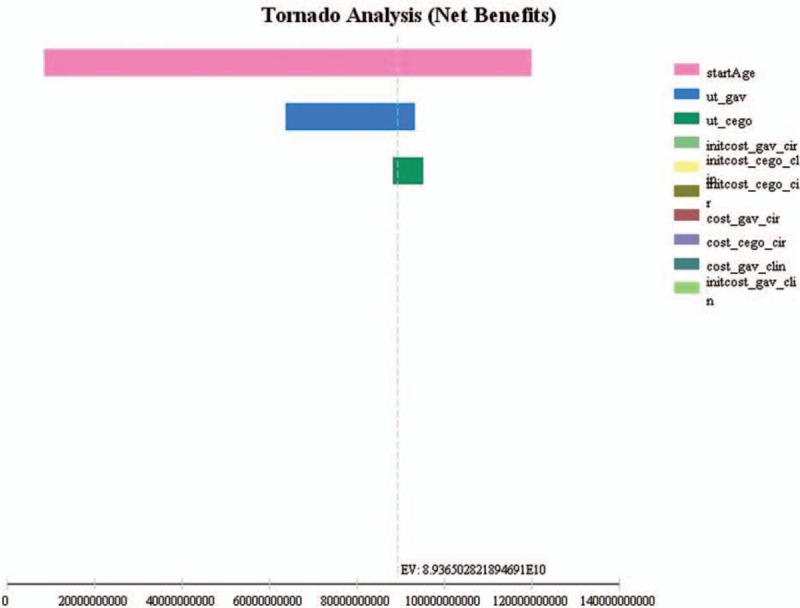
Model 3 (advanced glaucoma) sensitivity analysis (Tornado analysis) of all parameters.

We performed univariate sensitivity analysis for each model, including all model parameters. Figs. 1–3 show the results for the Tornado analysis. In all 3 models, the most influential parameter was the starting age, followed by the mean utility values. Cost variations did not have an impact on the results.

In model 1, 96.8% of all uncertainty is due to starting age. The younger the patient, the more cost-effective is the primary laser treatment. We observe no differences in cost-effectiveness profile for early glaucoma patients 70 years or older, for both primary laser and primary medications. Other variables had only minor impact on the results. Utility values for early and moderate glaucoma are responsible for 2.8% and 0.3% of the model 1 uncertainty. All other variables had almost no impact on the model results.

For the moderate glaucoma model, starting age also had a high impact on the results (96.6% of model uncertainty). In younger patients (<60 years), the laser treatment saves more money than in older patients. Age had little influence on cost-effectiveness ratios for surgery and medications. As in model 1, utility values had little impact on the results (moderate and advanced glaucoma utility values responsible for 3.3% and 0.1% of model 2 uncertainty).

Starting age was also an important variable for the advanced glaucoma model (explaining 93.1% of model uncertainty). The difference in cost-effectiveness ratios between the 2 treatment strategies are more evident in patients 70 years and younger (favoring surgery). The cost-effectiveness profile of both alternatives in advanced glaucoma patients over 70 is similar. Utilities also had little influence on our model results. They were responsible for 6.6% (advanced glaucoma utility value) and 0.4% (bilateral blindness utility value) of all model 3 uncertainty.

## 4 Discussion

Our results show that, from the SUS perspective and for patient life expectancy, for early glaucoma patients, both primary laser and primary medications are cost-effective. Both alternatives provide gains in quality of life (measured in QALY) when compared with observation (no treatment). We have also demonstrated that, for moderate glaucoma patients, all 3 options (medications, laser, and surgery as primary therapy) are cost-effective. However, laser and surgery provided the best cost-effectiveness profile, and medications led to higher costs. In advanced glaucoma patients, both medications and surgery were cost-effective options.

We decided to construct 3 models (early, moderate, and advanced glaucoma) to simulate glaucoma diagnosis over the whole spectrum of the disease. Ophthalmologists can diagnose patients in different stages of the disease. In Brazil, the great majority of patients are diagnosed in late glaucoma stages. We believe adapting treatment strategy to glaucoma stage is highly recommendable and can lead to cost savings.

The World Health Organization (WHO) classifies health interventions’ cost-effectiveness profiles according to each country's gross domestic product (GDP).^[32]^ An intervention is highly cost-effective when the cost per effectiveness gain (eg, US$/QALY gained) is less than 1 GDP per capita.^[32]^ It is still cost-effective when it does not exceed 3 times the GDP per capita.^[32]^ If it exceeds 3 times the value of GDP per capita, this intervention is not cost-effective.^[32]^ The GDP per capita in Brazil was R$ 27,299.00 (US$ 10,262.78) in 2014.^[33]^ Considering the 3-times GDP per capita threshold (R$ 81,897.00 or US$ 30,788.35), all glaucoma interventions for all 3 models were cost-effective. The highly cost-effective alternatives were laser and medications for early glaucoma; laser and surgery for moderate glaucoma; and surgery and medications for advanced glaucoma. The use of medications as primary therapy in moderate glaucoma patients can lead to high costs that are almost not cost-effective (R$ 80,568.09/QALY or US$ 30,288.76 / QALY).

For early glaucoma patients, primary laser provided the best cost-effectiveness ratio in comparison with medications (R$ 2811.39/QALY vs R$ 3450.47/QALY). In the sensitivity analysis, this difference is larger when the patient is younger. Glaucoma medications are responsible for the largest proportion of medical direct costs.^[3,4]^ The reason laser is more cost-effective at younger ages is that these patients do not need medications for some time after laser. Therefore, medical direct costs are lower for these patients.

Our study results for early glaucoma stage are not different from those published by Stein et al. They found that both primary laser therapy and primary treatment with prostaglandins were cost-effective in the United States.^[13]^ In this study, in a horizon of 25 years, ICER was US$ 16,824/QALY for laser and US$ 14,179/QALY for medical treatment.^[13]^ In our model, we have found lower values per QALY (US$ 1056.91/QALY for laser and US$ 1297.17/QALY for medications).

In Australia, researchers have developed a dynamic model to study the economic efficiency of initial laser therapy versus medical treatment for early-stage glaucoma.^[11,12]^ They have shown that initial laser treatment is a cost-saving alternative to the public health system.^[11,12]^ Cantor et al^[23]^ assessed the costs of 3 different glaucoma treatment strategies over a 5-year period. They compared laser trabeculoplasty, medications, and filtering surgery, and found that initiating glaucoma therapy with laser was the least expensive option.

For moderate and advanced glaucoma patients, filtering surgery appears as a highly cost-effective alternative, leading to lower costs and significant gains in quality of life when compared with the most common treatment in clinical practice (medications). Laser also appears as a good option in moderate glaucoma patients.

We can define a QALY as 1 year lived in perfect health.^[34]^ In model 1, observation-arm patients gained 10.2489 QALYs for the remainder of their lives. Patients submitted to both laser and medications experienced significant gains in QALY. Primary laser and primary medical therapy patients had an increment of approximately 2.5 QALYs and almost 5 QALYs, respectively, in comparison with no treatment. This ratifies the importance of the need for glaucoma treatment. The benefit of glaucoma treatment could extrapolate these gains in quality of life when societal costs (nonmedical direct and indirect costs) are included in the model. We encourage more research considering all glaucoma-related costs.

Starting age is a major driver for the cost-effectiveness analysis of glaucoma treatment strategies in any disease stage. Laser or surgery for younger patients (<70 years) lead to important cost-savings, mainly for postponing the use of glaucoma medications. The cost-effectiveness ratio is very similar among all treatment strategies for patients older than 70 years. Life expectancy can influence our results and we cannot extrapolate our results for other populations or countries with different life expectancies.

Our study suffers from some limitations. For lack of data, we did not stratify patients according to risk factors for glaucoma progression, such as race, central corneal thickness, corneal biomechanics, family history of blindness, perfusion pressure, and so on. We decided, in this model, to consider the average glaucoma patient. The availability of data and the adoption of assumptions may have influenced our results.

We obtained the transition probabilities from clinical trials in the literature. Clinical trial study patients are different from real-life patients because they are closely monitored to minimize patient loss and optimize adherence and persistence. We decided to use clinical trial data because there are no population studies in real life, which show the rate of progression and outcomes of the natural history of glaucoma (treated with different strategies vs untreated).

Another limitation is that our study did not allow patients in model 1 (observation vs laser vs medications) to go for filtering surgery as a rescue treatment. We also did not consider adherence and persistence in the medical therapy arm. This did not influence our results as patients in treatment at the SUS Glaucoma Referral Centers receive medications every 3 months (whether they have used them or not).

Finally, one should be very cautious when extrapolating our results for patients with other types of glaucoma and glaucoma patients under treatment outside the SUS setting.

In conclusion, all glaucoma treatment strategies were cost-effective by WHO guidelines. However, we could rank the most cost-effective alternatives for each glaucoma stage. The most cost-effective options for early glaucoma are, in order, primary laser and primary medications. In moderate glaucoma, surgery provides the best cost-effectiveness ratio, followed by laser and medications. For advanced glaucoma patients, surgery was the most cost-effective alternative (less expensive and presented almost the same effectiveness as medical therapy). Medical treatment was also cost-effective for advanced glaucoma.
